# The Transmembrane Isoform of *Plasmodium falciparum* MAEBL Is Essential for the Invasion of *Anopheles* Salivary Glands

**DOI:** 10.1371/journal.pone.0002287

**Published:** 2008-05-28

**Authors:** Fabian E. Saenz, Bharath Balu, Jonah Smith, Sarita R. Mendonca, John H. Adams

**Affiliations:** 1 Global Health and Infectious Diseases Research, Department of Global Health, College of Public Health, University of South Florida, Tampa, Florida, United States of America; 2 Department of Biological Sciences, University of Notre Dame, Notre Dame, Indiana, United States of America; Federal University of São Paulo, Brazil

## Abstract

Malaria transmission depends on infective stages in the mosquito salivary glands. *Plasmodium* sporozoites that mature in midgut oocysts must traverse the hemocoel and invade the mosquito salivary glands in a process thought to be mediated by parasite ligands. MAEBL, a homologue of the transmembrane EBP ligands essential in merozoite invasion, is expressed abundantly in midgut sporozoites. Alternative splicing generates different MAEBL isoforms and so it is unclear what form is functionally essential. To identify the MAEBL isoform required for *P. falciparum* (NF54) sporozoite invasion of salivary glands, we created knockout and allelic replacements each carrying CDS of a single MAEBL isoform. Only the transmembrane form of MAEBL is essential and is the first *P. falciparum* ligand validated as essential for invasion of *Anopheles* salivary glands. MAEBL is the first *P. falciparum* ligand experimentally determined to be essential for this important step in the life cycle where the vector becomes infectious for transmitting sporozoites to people. With an increasing emphasis on advancing vector-based transgenic methods for suppression of malaria, it is important that this type of study, using modern molecular genetic tools, is done with the agent of the human disease. Understanding what *P. falciparum* sporozoite ligands are critical for mosquito transmission will help validate targets for vector-based transmission-blocking strategies.

## Introduction

*Plasmodium falciparum* is the malaria parasite responsible for several million deaths each year. Mosquitoes of the genus *Anopheles* are the vectors of this parasite and consequently play a crucial role in the transmission of malaria. Nevertheless, the biology of the mosquito stages of *P. falciparum* has been poorly studied and few of the essential molecular interactions are identified. After a mosquito takes a blood meal, gametes quickly mature in the mosquito and fertilize to become a motile zygote, or oökinete, that invades the midgut of the mosquito, and develops into an oocyst where sporozoites form for a period of 10 days to 2 weeks [1], [2]. The sporozoites are released in the hemolymph and relatively few manage to invade the distal and medial lobes of the salivary gland [3].

Invasion of the salivary glands is a two-stage process that requires traversal of the basal lamina and then entry into the salivary gland cell [1]. Similar to merozoite invasion of erythrocytes, invasion of salivary glands occurs in several steps: initial attachment, reorientation, junction formation and entry via a moving junction [1]. Like the merozoite, the sporozoite has organelles, such as the micronemes and the rhoptries, which have proteins to mediate invasion of the host cells [4].

Three *Plasmodium* microneme proteins have been shown to be important for sporozoite transmission through the mosquito vector using rodent malaria models. Circumsporozoite protein (CSP) is a GPI-anchored protein essential both for oocyst development and sporozoite invasion of the salivary glands [5], [6]. Thrombospondin anonymous protein (TRAP) is a transmembrane protein required for gliding motility of sporozoites and invasion of but not initial attachment to salivary glands [7], [8]. Mutant parasites lacking the cytoplasmic domain of TRAP are not infective to the liver cells and do not show gliding motility [8]. These functions of TRAP depend on its cytoplasmic domain, which is homologous to the cytoplasmic domain of the *Toxoplasma* microneme protein-2, and its interactions with the glideosome. It is well documented that interactions of these cytoplasmic domains with the glideosome motor complex are mediated via molecular bridging with aldolase [9], [10].

MAEBL, a paralogue of erythrocyte binding proteins (EBP) [11]–[13] known to be essential for merozoite invasion of erythrocytes, is a third transmembrane ligand that has been shown to be important in the process of salivary gland invasion [14]. Importantly, the EBP family ligands share structural homology with MAEBL in the extracellular carboxyl cysteine-rich domain and in the presence of transmembrane and cytoplasmic domains, but do not appear to interact with the glideosome in the same way as TRAP or its homologues. Amino terminal cysteine-rich domains of MAEBL have similarity to the Apical membrane antigen-1 (AMA1) ectodomain and exhibit cytoadhesive properties in vitro [12], [15]. MAEBL is highly expressed in midgut sporozoites and on the surface of salivary glands sporozoites [16]–[18]. In *Plasmodium berghei*, MAEBL was found to be essential for attachment and invasion of salivary glands [14].

Expression of MAEBL is developmentally regulated in the midgut and salivary gland sporozoite with the production of two dominant alternatively spliced isoforms [18]. This alternative splicing of the 3′ exons generates coding sequences (CDS) for a secreted MAEBL isoform in addition to the transmembrane MAEBL isoform. The two main isoforms of MAEBL are referred to as ORF1 (with C-terminal transmembrane domain) and ORF2 (without C-terminal transmembrane domain). The ORF1 product is structurally homologous to the Duffy binding-like erythrocyte binding protein (DBL-EBP) ligands. ORF2 is a putative soluble isoform that has an alternative carboxyl terminus without a transmembrane domain and its accompanying cytoplasmic domain. ORF1 and ORF2 are differentially expressed in midgut and salivary gland sporozoites and MAEBL expression was best correlated with the appearance of the canonical ORF1 transmembrane transcript [18].

Because of this complexity in expression it is not clear what isoform of MAEBL is functionally important in mature sporozoites for invasion of the salivary glands. Either MAEBL serves simply as an adhesin to mediate sporozoite attachment to the salivary glands or the C-terminal portion of MAEBL also has a role to interact with cytoplasmic elements to mediate its function during invasion. By studying the role of the different forms of MAEBL it is possible to understand better the biology of the *Plasmodium* sporozoite and, as a result, generate information useful for the development of tools such as a transmission blocking vaccine to stop the parasite passage in the vector. Here we show that a transmembrane form of *P. falciparum* MAEBL is essential for the invasion of the *Anopheles* salivary glands.

## Results

### MAEBL knockout and allelic replacements

**Figure 1:**
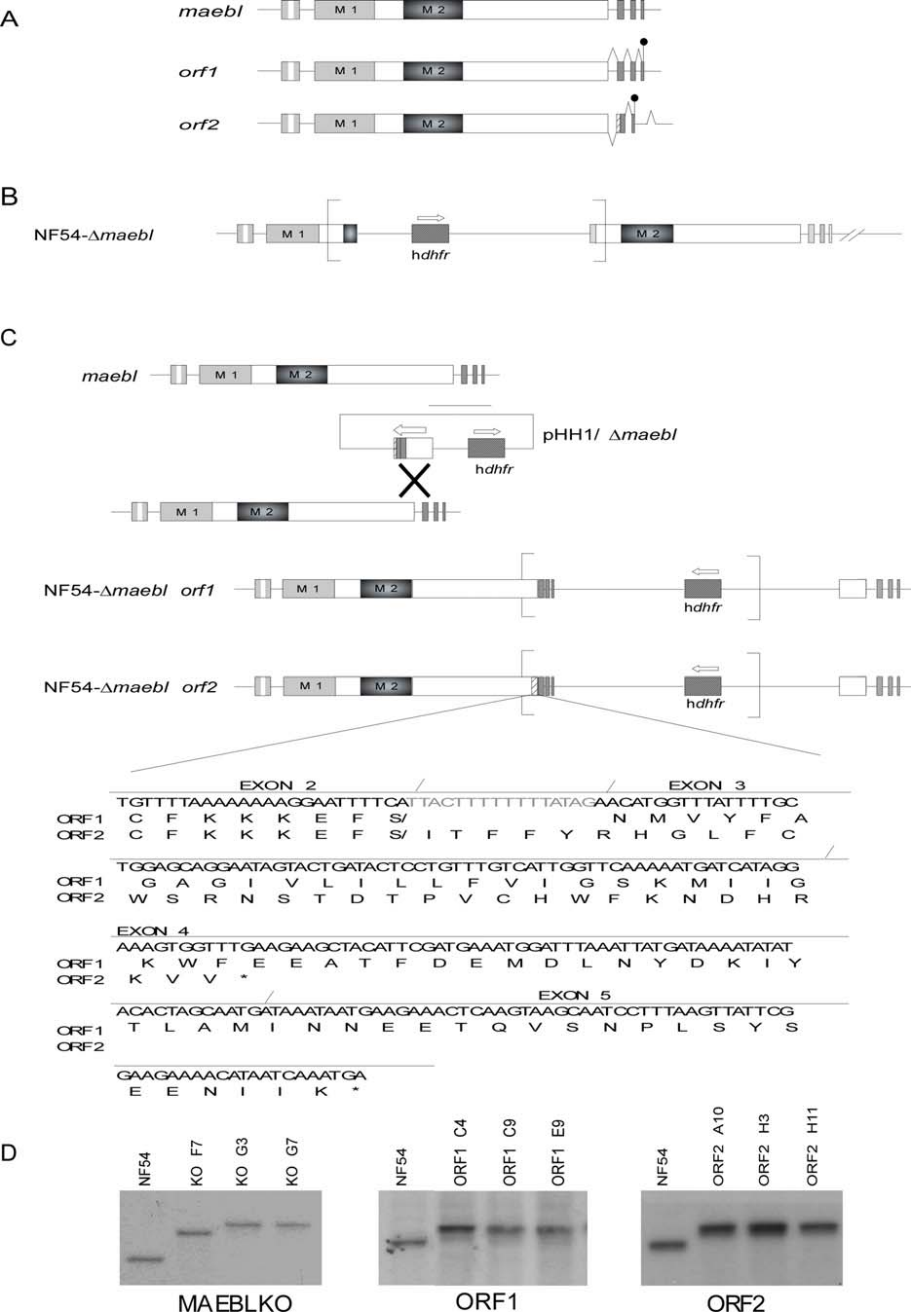
Allelic replacement of the carboxyl terminal end of *maebl.* A. Alternative splicing pattern of *maebl* gene [18]. The two main splicing patterns of *maebl* are shown. ORF1 encodes the canonical, transmembrane form of MAEBL, and consists of 5 exons. ORF2 is produced by a shift in the ORF that encodes a distinct coding sequence with a hydrophilic C-terminus, changing the canonical type I transmembrane MAEBL product to a soluble isoform with a stop at the second codon in exon 4 resulting in early termination of the CDS that is 50 nt before the next intron. B. Schematic representation of the disruption of the 5′ end of *P. falciparum maebl* using the vector pHD22YΔMAEBL that has a human *dhfr* selectable marker. C. Schematic representation of the allelic replacement of the 3′ end of *P. falciparum maebl* using the vector pHH1ΔMAEBL that has a human *dhfr* selectable marker. The 3′ end of *maebl* with the cDNA sequence corresponding to ORF1 or ORF2 was cloned into the pHH1 vector. The alignment shows the nucleotide and amino acid sequences of ORF1 and ORF2. ORF2 has 16 extra nucleotides that cause a shift in the ORF switching from hydrophobic to hydrophilic amino acids and an early stop in exon 4. D. Screening for transformed clones by Southern blot hybridization analyses of genomic DNA. Transformed drug resistant parasites identified plasmid integration into the *maebl loc*us in drug resistant populations. ORF1 mutant clones C4, C9 and E9 show the allelic replacement at the 3′ end. ORF2 *clones* A10, H3, H11 show the allelic replacement at the 3′ end. C. MAEBLKO mutant clones F7 G3 and G7 show plasmid integration between the M1 and M2 domains.

Targeted integrations at the *maebl* locus were achieved in *P. falciparum* NF54 by single homologous recombination events. Two different allelic replacements of the 3′ end of *maebl* were created in NF54 parasites to express either the ORF1 or ORF2 isoform. Multiple independent clones were obtained as ORF1-only (ORF1) or ORF2-only (ORF2) MAEBL expression mutants. Three independent *maebl* knockout clones were also created as negative controls and NF54 parental parasites were used as positive controls for invasion of the mosquito salivary glands. The expected integration into the *maebl* locus was confirmed for each mutant **(** Figure 1). There were no differences in blood-stage growth or the erythrocyte invasion phenotype for any of the genetically mutated parasites. Growth assays in neuraminidase, trypsin and chemotrypsin treated red blood cells using MAEBL mutant parasites did not show any significant difference with the parental clone NF54 (data not shown). In addition, all the parasites were able to produce gametocytes that matured and formed male and female gametes similar to the parental clone NF54 (data not shown).

### Mosquito infections: oocyst and midgut sporozoites

*Anopheles freeborni* and *An. stephensi* mosquitoes were fed mature *P. falciparum* NF54 gametocytes (days 15 and 16) using a biological membrane feeding system. Infections in the midgut, hemolymph and salivary glands at days 10, 18, and 20 post blood meal feeding, respectively, were identified by microscopic observation in a Nomarski bright field microscope and by fluorescence microscopy using dihydroethidium as a nuclear stain. In addition, transcript analysis for *csp* was used to confirm the presence or absence of sporozoite infections in each of the mosquito organs. An average of 70% of the midguts were infected and we obtained an average of 9 oocyst per mosquito midgut.

**Figure 2:**
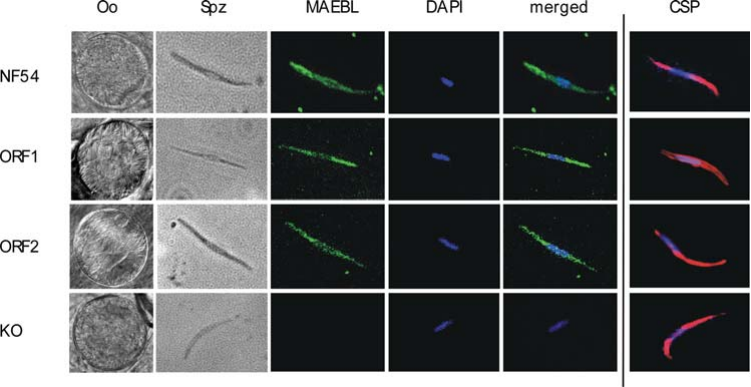
ORF1 and ORF2 parasites express MAEBL in midgut sporozoites. ORF1, ORF2, and MAEBL KO in *Anopheles* midgut oocysts (Oo) and sporozoites (Spz) are shown in Nomarski bright field having similar phenotypes to the parental clone NF54. Midgut sporozoites from NF54 parasites, MAEBL KO, ORF1, and ORF2 were purified as previously described [7] and incubated with M2sd2 antibody (against the M2 ligand domain of MAEBL) and DAPI (nuclear stain). ORF1 and ORF2 sporozoites show a similar expression of MAEBL as the parental clone NF54. MAEBL appears to be distributed throughout the body and on the surface of ORF1 and ORF2 sporozoites. As expected, MAEBL KO sporozoites do not express MAEBL. All the genetically modified parasites express CSP on the surface of the sporozoite similar to the parental clone NF54.

The presence of oocysts in the anopheline midguts was confirmed on day 10 post blood meal feeding. The MAEBL mutant parasites produced morphologically and developmentally normal, or wild type (WT), oocysts in the midguts of *Anopheles* mosquitoes (Figure 2). The oocysts of the genetically modified parasites typically required 10–14 days to mature and sporozoites were observed budding from sporoblasts similar to the parental clone NF54 (Figure 2). Quantitative analysis using a Student's *t*-test indicated there were no significant differences in the number of infected midguts among the mutant clones relative to WT NF54 parasites (P value NF54 vs MAEBL KO: P (0.2962), NF54 vs ORF1: P(0.6018), NF54 vs ORF2, P(0.0902) or in the number of oocysts per midgut (P value NF54 vs MAEBL KO: P (0.1428), NF54 vs ORF1: P(0.1508), NF54 vs ORF2, P(0.5622) (Table 1). Microscopic observation of the hemolymph of infected mosquitoes confirmed the presence of sporozoites for all the mutant parasites, including the MAEBL knockout lines (MAEBL KO, ORF1, ORF2). Indeed, sporozoites released into the hemolymph from the oocysts of mutant parasites appeared similar in abundance and morphology (Table 1).

**Table 1:** Number of *P. falciparum* infections in *Anopheles freeborni* females.

Parasite (clone)	Midgut oocysts	Midgut oocysts	Midgut oocysts	Hemolymph sporozoites	Hemolymph sporozoites	Salivary gland sporozoites	Salivary gland Sporozoites
	n	Av (SE)	%	Av (SE)*	%+	Av (SE)	%+
NF54	289	9 (2.1)	72	20.2 (16.1)	67	701 (129.7)	85
ORF1 (C9, E9)	224	14.5 (3.3)	68	15.2 (11.8)	75	565 (150.8)	89
ORF2 (A10, H11)	144	7.5 (1.3)	61	20 (17.7)	83	0	0
MAEBL KO (G3,G7)	154	5.5 (2.6)	78	22.6 (24.0)	60	0	0

* Pulled from 8 mosquitoes

+ Percentage of infected mosquitoes

Normal expression patterns of MAEBL were confirmed in mutant parasites by RT-PCR (data not shown) and by IFA (Figure 2). Immunofluorescence localization patterns in the ORF1 and ORF2 allele-specific parasites, using antibodies against the M2 ligand domain of MAEBL, were similar to NF54. For example, midgut sporozoites in day 13 of NF54, ORF1-specific mutants and ORF2-specific mutants showed distribution of MAEBL on the surface of the sporozoite (Figure 2). As expected, MAEBL KO sporozoites did not express MAEBL whereas CSP immunofluorescence patterns were similar in NF54 and all the mutant parasites, including the MAEBL knockout. These results suggest that the localization of MAEBL in ORF2, ORF1 and NF54 parasites is the same in spite of the structural differences in MAEBL proteins.

### Sporozoites in the salivary glands

**Figure 3:**
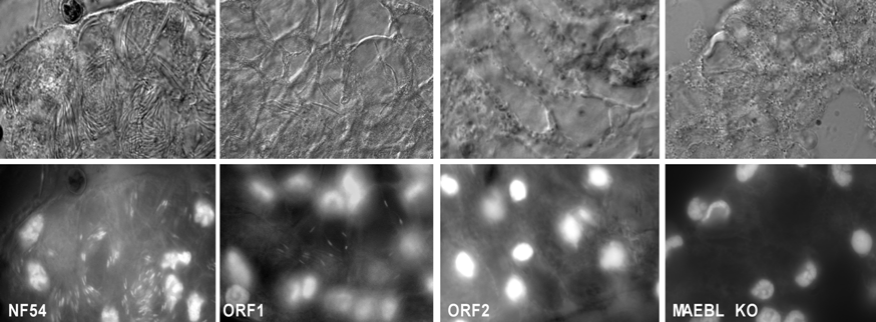
Sections of salivary glands of *Anopheles* mosquitoes infected with NF54, ORF1, ORF2 and MAEBL KO on day 19 post feeding are shown in Nomarski bright field images (top lane) and by nuclear fluorescence using dihydroethidium (bottom lane). The sporozoites can be seen as small nuclei inside the salivary cells. NF54 and ORF1 sporozoites are visible inside salivary glands cells whereas no sporozoites of ORF2 or MAEBL KO clones were found in salivary glands. These results were confirmed by RT-PCR using primers for *csp*.

**Figure 4:**
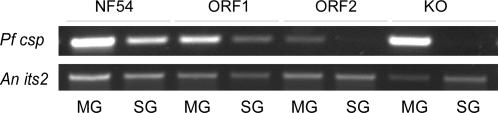
RT-PCR analyses determines that ORF1 parasites invade salivary glands cells. RT-PCR analyses of NF54, ORF1, ORF2, and MAEBL KO parasites in *Anopheles* midgut (MG) day 10 and *Anopheles* salivary glands (SG) day 20. Primers specific for *P. falciparum csp* were used to detect the presence of parasites and *Anopheles its2* were used as controls. A band is observed in the midguts and salivary glands of the NF54 and ORF1 infected *Anopheles*. While the midguts of ORF2 or MAEBL KO infected mosquitoes are positive, the salivary glands were RT-PCR negative for detection of the *P. falciparum csp*.

*P. falciparum* sporozoites usually begin to infect the salivary glands of *Anopheles* mosquitoes beginning day 14 post blood meal. This was confirmed in NF54 parasites, where hundreds or thousands of sporozoites were observed inside the salivary glands (Figure 3). Similar to the wild type NF54, ORF1 mutant parasites had abundant sporozoites in the salivary glands (Figure 3, Figure S1, Table 1). In contrast, no sporozoites were observed or detected in the salivary glands of mosquitoes fed the MAEBL KO parasites or ORF2 mutant parasites (Figure 3), even though midgut sporozoites were readily observed beginning day 10 in the hemolymph of these mosquitoes infected with the MAEBL KO or ORF2 mutant. The absence of sporozoites in the salivary glands determined by microscopic analysis was confirmed with RT-PCR using primers for *csp*, the most highly expressed gene in sporozoites (Figure 4).

## Discussion

Sporozoite invasion of the anopheline salivary gland is critical for malaria parasite transmission and yet it is one of the most poorly studied stages of this deadly pathogen. In this study we provide the first characterization of a *P. falciparum* ligand essential for the invasion of the mosquito salivary glands. The critical ligand is MAEBL, which is a paralogue of EBA-175 and other erythrocyte binding proteins known to mediate an essential step for merozoite invasion of erythrocytes. Since alternative splicing of *maebl* is conserved among all *Plasmodium* species examined [18], it is not possible to know from previous studies what MAEBL isoform is essential for invasion [14]. We find that only the transmembrane isoform of MAEBL (ORF1) is essential for the invasion of salivary glands, indicating an involvement of the cytoplasmic domain of MAEBL in the invasion process.

No MAEBL knockout and ORF2 mutant sporozoites were present inside the salivary gland cells and sporozoites were not arrested in the invasion process on the surface of the glands. This observation that the MAEBL deficient mutants cannot attach to salivary glands coincides with previous studies [14] and contrasts with the data for TRAP in which TRAP deficient sporozoites were found on the surface of the salivary glands, but not inside the salivary cells [7]. Importantly, sequence analysis (Figure S2) shows that the MAEBL cytoplasmic domain lacks the penultimate tryptophan flanked by acidic residues at its C-terminus (Figure S2), which are known to be essential for TRAP binding to the parasite's glideosome complex via aldolase [8], [12], [19]. Although we found that there is an acidic motif near the C-terminus of *P. falciparum* MAEBL, these terminal residues are not fully conserved among MAEBL products of other *Plasmodium* species [12]. The absence of these critical residues in the MAEBL cytoplasmic domain suggests that if this ligand interacts with aldolase or other components of the glideosome then this interaction has a molecular basis different from the TRAP-aldolase interaction.

Based on its homology to the EBPs, which are critical for junction formation during merozoite invasion of erythrocytes, MAEBL may be important in a similar step for sporozoites invasion into the salivary gland cell. AMA1 has a major role in *P. falciparum* merozoite attachment and reorientation to erythrocytes [20] and its high level of expression in sporozoites [21] suggests that it may have a simi zlar role in the sporozoite invasion process. Similarly, MAEBL ligand domains and AMA1 ectodomain sequence and structure analysis comparison suggests a role in similar steps in the invasion of host cells [22].

This would place the function of MAEBL before TRAP involvement during the sporozoite invasion process into salivary glands. Therefore, we conclude that we do not see sporozoites attached to the salivary glands for the MAEBL deficient mutants, because similar to merozoite's initial attachment the sporozoite attachment to salivary glands is an unstable and reversible step when invasion does not proceed to junction formation. These results are consistent for *P. falciparum* MAEBL to have a role as an essential ligand in the early stages of the invasion process.

The fact that a transmembrane form of MAEBL is essential in the process of invasion of salivary glands validates the importance of alternative splicing in *Plasmodium*. As in higher eukaryotes, alternative splicing may be an under appreciated mechanism for the expression of different malaria parasite products that could be generating product diversity [23], [24]. At this point we can only speculate about the function of ORF2, which may be participating in a different phase of the interactions with the host cell or alternative splicing may be participating in the regulation of gene expression through a nonsense mediated decay mechanism [25], [26].

Transmission of malaria is dependent upon successful completion of parasite development in the mosquito. Here we show that the *P. falciparum* MAEBL transmembrane protein is essential for the infective stages to enter the salivary glands and this role appears to be different from the role of TRAP. In addition, our study demonstrates the feasibility of using genetically modified *P. falciparum* to study host-parasite interactions in the vector stages of development. The essential role of MAEBL for the sporozoite invasion of the mosquito salivary gland is a weakness in the parasite's biology that provides a potential opportunity for vector-based intervention strategies to disrupt malaria transmission.

## Materials and Methods

### Plasmids Construction

For MAEBL-KO clones, a plasmid construct for single homologous recombination was created by using the plasmid pHD22Y [27]. A 750 bp region of *maebl* was amplified from the *P. falciparum* NF54 genomic DNA by using primers JA-1410 (5′ AGATCTCCAACATGTGTACTGAAAAAGG 3′) and JA-1411(5′ CTCGAGCCTAGAAGATTTTGTACAATAATAGC 3′). The conditions for PCR were 94°C for 1 min followed by 35 cycles of 15 s at 94°C; 30 s at 49°C; 1 min at 65°C. The PCR product was cloned into pGEM-T easy vector (Promega) and excised as an *EcoR* I fragment and cloned into the vector pHD22Y. For MAEBLΔORF1 and MAEBLΔORF2 the plasmids constructs for single homologous recombination were created by using the plasmid pHH1 [28]. A 1100 bp Region of the 3′ end of MAEBL was amplified from the *P. falciparum* NF54 cDNA using the primers JA-1441 (5′ AGATCTGAAGATGAAAAAAGAATGGAAGTA 3′) and JA-1442 (5′ CTCGAGCAAAAAAAATAAACCCACAAAAAAGTAC 3′). The conditions for RT-PCR were 45°C for 30 min 94°C for 2 min followed by 35 cycles of 15 s at 94°C; 30 s at 48°C; 1 min at 65°C. The PCR products were cloned into a pGEM-T easy vector (Promega), sequenced to identify ORF1 and ORF2 DNA and excised as a *Bgl II XhoI* fragment. The fragments were cloned into the vector pHH1 to make the pHH1-ORF1 and pHH1-ORF2 plasmids.

### Parasites and transformations

*P. falciparum* parasites were grown in human A+ erythrocytes as previously described [29]. The plasmids were transfected by homologous recombination into erythrocytes and then purified merozoites were used to invade the erythrocytes as previously described [30]. Positive selection for transfectants was achieved using 5 nM WR99210, an antifolate that selects for the presence of the human *dhfr* gene [27]. Southern blot hybridizations were performed using standard protocols to confirm integration in the *maebl* locus [11]. Two micrograms of genomic DNA from *P. falciparum* NF54 [31] and Δ*maebl* clones was digested with *Eco*Rv or NsiI. The restricted fragments were separated by 0.8% agarose gel, transferred to nylon membranes and hybridized with ^32^P-labeled probes of *hDHFR* and *maebl* M2 domain.

### Determination of parasite red blood cells invasion phenotype

Washed erythrocytes were treated with either 1 mg/ml trypsin (TPCK treated) (Sigma), 1 mg/ml chymotrypsin type-VII (Sigma), or 0.5 Unit/ml neuraminidase type II (Sigma) for 1 hr at 37°C with gentle shaking, followed by treatment with the appropriate protease inhibitor for 10 min. Erythrocytes were then washed with RPMI1640 (Gibco). Mature schizonts from *P. falciparum* NF54 wild-type and *maebl* mutant clones were purified by passage through a magnetic column (Miltenyi Biotech). The purified schizonts were allowed to invade normal and enzymatically treated erythrocytes. The cultures were initiated in a 96-well plate at 1% parasitemia with 1% hematocrit in low hypoxanthine media. After 20 hrs of growth, 0.5 µCi ^3^H was added to the cultures. Cultures were incubated overnight, and the culture plate was frozen at −80°C for 4hr. The cells were harvested on filter (filtermat), a nd were counted by a Microbeta Plate 1450 (Perkin Elmer).

### Production of gametocytes and passage through mosquitoes

Asexual and mature gametocytes NF54 isolate and NF54 transformed parasites were cultivated in vitro as previously described [32]. Mosquitoes used in these studies were *An. freeborni* and *An. stephensi* originally obtained from MR4, MRA-130 and MRA-128, respectively. Mosquito stages (oocysts, hemolymph, and salivary glands) were obtained after membrane feeds of on blood containing mature gametocytes as described previously [33].

### Mosquito dissections and collection of sporozoites

The *Anopheles* mosquitoes were dissected on days 10 and 18 post feed for midguts which were stained with dihydroethidium (Molecular Probes) to make easier the identification of oocysts and sporozoites. The hemolymph was collected on day 18 post feed by spinning down mosquito thoraxes without salivary glands or by injecting medium 199 (Invitrogen) and collecting the first drops from the abdomen, as described in [7]. The collected liquid was mixed with dihydroethidium in order to identify facilitate locating the sporozoites. The anopheline salivary glands were dissected on day 20–22 and stained with dihydroethidium. The presence or absence of spororozoites was recorded.

### Analysis of the expression of MAEBL in mosquito stages

*RT-PCR transcript analysis*. Infected midguts and salivary glands were crushed and processed for RNA as previously described [18]. The RNA was converted to cDNA (Invitrogen super script 2) and then amplified with primers for *maebl* M2 subdomain 1, JA 1484 (5′ AAACCCGCAACAAAAATATATGGAAAGATT 3′) and JA 1949 (5′ AAGCTTTAAAGGGTATCTAGGTGGACATTT 3′) and the carboxy terminal end of *maebl* JA 412 (5′ GTGATTATATGAAGGATAATATTTCATCAG 3′) and JA 471 (5′ CCGAATAACTTAAAGGATTGCTTAC 3′). *csp* was amplified using the primers 5′-GCTAATGCCAACAGTGCTGTA-3′ and 5′-GGAACAAGAAGGATAATACC-3′ and *Anopheles* rDNA internal transcribed spacer 2 (*its2*) was amplified using the primers 5′-TGTGAACTGCAGGACACAT-3′ and 5′-TATGCTTAAATTCAGGGGGT-3′.

### Indirect immunofluorescent assay

*P. falciparum* midgut sporozoites day 13 post feed were smeared on glass slides and air-dried. Slides were fixed in 1% formaldehyde, pre-incubated with 3% BSA 1% Triton in PBS, and then reacted with primary antibody (MAB 2C11) [13] and sera M2Sd2, or CSP sera). Secondary antibody was FITC-conjugated goat anti-mouse (Jackson Immuno Research Laboratories) or FITC-conjugated goat anti-rat IgG antibody (Jackson Immuno Research Laboratories). The slides were mounted and viewed by fluorescent microscopy and brightfield DIC microscopy; images were digitally recorded (Zeiss AxioCam) and further processed by 2D deconvolution software (Zeiss Axiovision).

## Supporting Information

Figure S1 ORF1 mutant parasites invade salivary glands cells. Sections of salivary glands of Anopheles mosquitoes infected with ORF1 on day 19 post feeding are shown. S1A. Nomarski bright field. S1B. Fluorescence image after dihydroethidium stain. The nuclei of the sporozoites and the nuclei of the salivary gland cells appear in the same focal plan indicating that the sporozoites are inside the salivary glands.
(1.60 MB TIF)

Figure S2 Comparative alignment of the cytoplasmic domains of invasion ligands: P. berghei AMA1, P. falciparum TRAP, P. berghei MAEBL, P. falciparum MAEBL and P. falciparum EBA175. The cytoplasmic domain of TRAP, MAEBL, and EBA175 are highly acidic (acidic residues shown in red). A TRAP terminal tryptophan has been shown to be essential form the invasion of salivary glands, this residue is not conserved in AMA1, MAEBL, and EBA175. AMA1 and MAEBL share a tryptophan residue in the cytoplasmic tail (shown in green boxes) but far from the penultimate position and are not immediately flanked by acidic residues. The terminal acidic residues that have been shown to be essential for the invasion of salivary glands in TRAP [1] are not conserved in MAEBL, AMA-1 or EBA-175 (boxed in grey). Tyrosine residues are shown in yellow boxes. 1. Kappe S, Bruderer T, Gantt S, Fujioka H, Nussenzweig V, et al. (1999) Conservation of a gliding motility and cell invasion machinery in Apicomplexan parasites. J Cell Biol 147: 937-944.
(0.09 MB TIF)
