# Supplementary figures and images for: The Transmembrane Isoform of Plasmodium falciparum MAEBL Is Essential for the Invasion of Anopheles Salivary Glands
Source: PLoS One. 2008 May 28;3(5):e2287. doi: 10.1371/journal.pone.0002287 (PMC2386256; doi:10.1371/journal.pone.0002287)

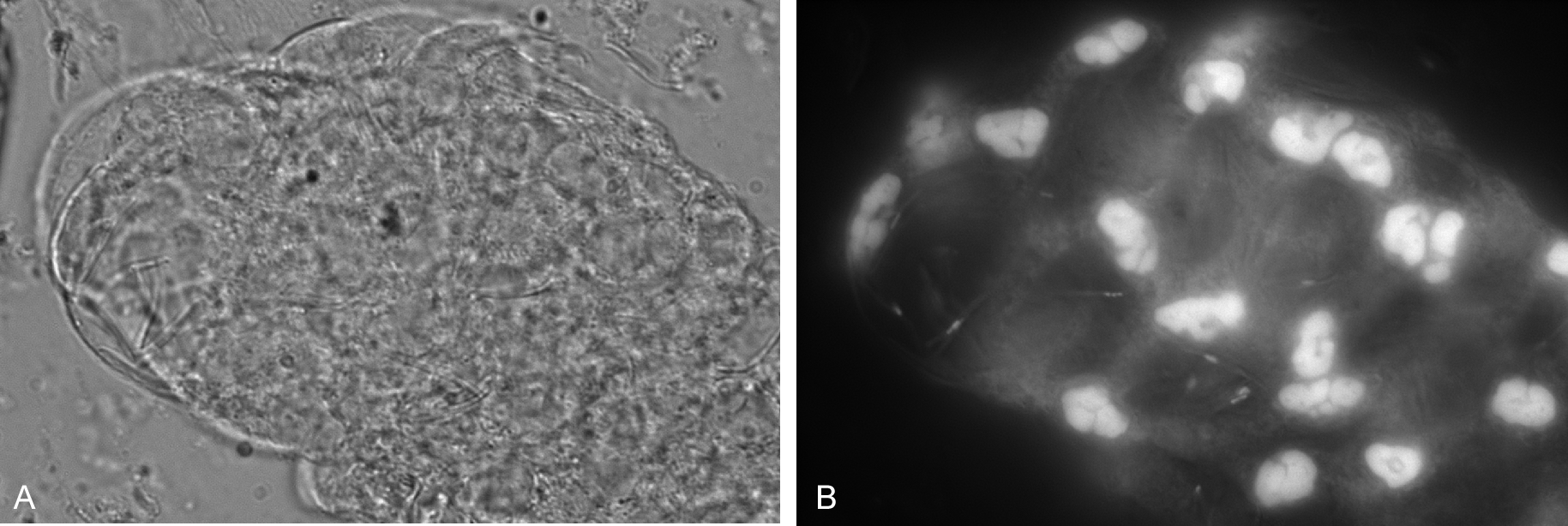

Supplement: Figure S1 — ORF1 mutant parasites invade salivary glands cells. Sections of salivary glands of Anopheles mosquitoes infected with ORF1 on day 19 post feeding are shown. S1A. Nomarski bright field. S1B. Fluorescence image after dihydroethidium stain. The nuclei of the sporozoites and the nuclei of the salivary gland cells appear in the same focal plan indicating that the sporozoites are inside the salivary glands. (1.60 MB TIF) [file pone.0002287.s001.tif]

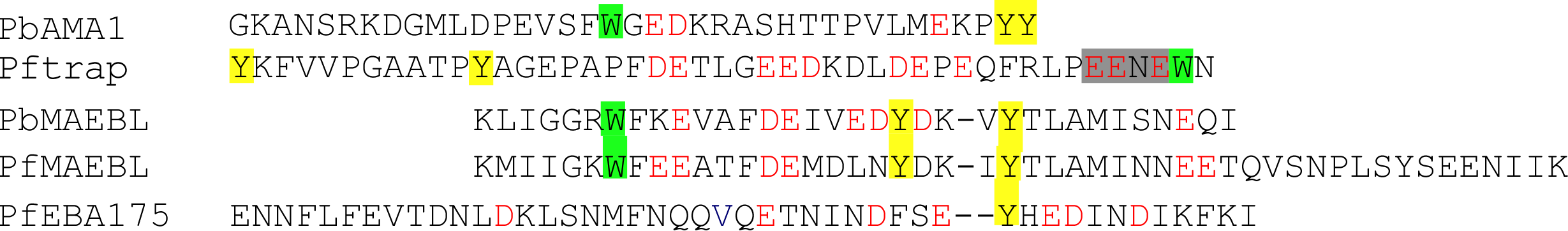

Supplement: Figure S2 — Comparative alignment of the cytoplasmic domains of invasion ligands: P. berghei AMA1, P. falciparum TRAP, P. berghei MAEBL, P. falciparum MAEBL and P. falciparum EBA175. The cytoplasmic domain of TRAP, MAEBL, and EBA175 are highly acidic (acidic residues shown in red). A TRAP terminal tryptophan has been shown to be essential form the invasion of salivary glands, this residue is not conserved in AMA1, MAEBL, and EBA175. AMA1 and MAEBL share a tryptophan residue in the cytoplasmic tail (shown in green boxes) but far from the penultimate position and are not immediately flanked by acidic residues. The terminal acidic residues that have been shown to be essential for the invasion of salivary glands in TRAP [1] are not conserved in MAEBL, AMA-1 or EBA-175 (boxed in grey). Tyrosine residues are shown in yellow boxes. 1. Kappe S, Bruderer T, Gantt S, Fujioka H, Nussenzweig V, et al. (1999) Conservation of a gliding motility and cell invasion machinery in Apicomplexan parasites. J Cell Biol 147: 937-944. (0.09 MB TIF) [file pone.0002287.s002.tif]
